# Assessment of the Steering Precision of a Hydrographic Unmanned Surface Vessel (USV) along Sounding Profiles Using a Low-Cost Multi-Global Navigation Satellite System (GNSS) Receiver Supported Autopilot

**DOI:** 10.3390/s19183939

**Published:** 2019-09-12

**Authors:** Mariusz Specht, Cezary Specht, Henryk Lasota, Piotr Cywiński

**Affiliations:** 1 Department of Transport and Logistics, Gdynia Maritime University, Morska 81-87, 81-225 Gdynia, Poland; 2 Department of Geodesy and Oceanography, Gdynia Maritime University, Morska 81-87, 81-225 Gdynia, Poland; c.specht@wn.umg.edu.pl; 3 Department of Marine Electronic Systems, Gdańsk University of Technology, 11/12 Gabriela Narutowicza, 80-233 Gdańsk, Poland; henlasot@pg.edu.pl (H.L.); peter@navinord.com (P.C.); 4 NAVINORD Piotr Cywiński, Długa 47, 83-260 Kaliska, Poland

**Keywords:** Unmanned Surface Vessel (USV), multi-Global Navigation Satellite System (GNSS) receiver, bathymetric measurements, cross track error (XTE)

## Abstract

The performance of bathymetric measurements by traditional methods (using manned vessels) in ultra-shallow waters, i.e., lakes, rivers, and sea beaches with a depth of less than 1 m, is often difficult or, in many cases, impossible due to problems related to safe vessel maneuvering. For this reason, the use of shallow draft hydrographic Unmanned Surface Vessels (USV) appears to provide a promising alternative method for performing such bathymetric measurements. This article describes the modernisation of a USV to switch from manual to automatic mode, and presents a preliminary study aimed at assessing the suitability of a popular autopilot commonly used in Unmanned Aerial Vehicles (UAV), and a low-cost multi-Global Navigation Satellite System (GNSS) receiver cooperating with it, for performing bathymetric measurements in automated mode, which involves independent movement along a specified route (hydrographic sounding profiles). The cross track error (XTE) variable, i.e., the distance determined between a USV’s position and the sounding profile, measured transversely to the course, was adopted as the measure of automatic control precision. Moreover, the XTE value was statistically assessed in the publication.

## 1. Introduction

Hydrography is a branch of applied science which deals with the measurement and description of physical features of the navigable portion of the Earth’s surface and adjoining coastal areas, with special reference to their use for the purpose of navigation [1]. The basic activity of national hydrographic institutions is the creation of nautical charts based on measurement data obtained from direct depth measurements performed by hydrographic vessels. The precision of steering an unmanned hydrographic vessel along sounding profiles with the aim of measuring the depth and creating a bathymetric chart of an inland or marine waterbody is the main factor determining the quality of the bathymetric chart under creation. In traditional (manned) solutions, a vessel is controlled manually or in automatic mode using a vessel autopilot. The methodology of performing such measurements has been described comprehensively and in detail in the literature on the subject [2,3,4,5] and includes the following issues and processes: the selection of technical equipment for the vessel [6,7], planning of measurements [8], their performance [9,10], and the processing of results [11,12,13,14].

Contemporary bathymetric measurements are performed in a traditional (manned) manner using complex bathymetric systems based on MultiBeam EchoSounders (MBES) [15]. This enables the simultaneous measurement of a wide swath of the seabed, and Global Navigation Satellite Systems (GNSS) with various positioning accuracy, starting from the marine Differential Global Positioning System (DGPS) ensuring a positioning accuracy of 1–2 m (*p* = 0.95) [16,17], through to the European Geostationary Navigation Overlay Service (EGNOS), with an accuracy of 2–3 m (*p* = 0.95) [18,19], ending with solutions based on GNSS geodetic networks ensuring an accuracy of 2–5 cm (*p* = 0.95).

**Figure 1:**
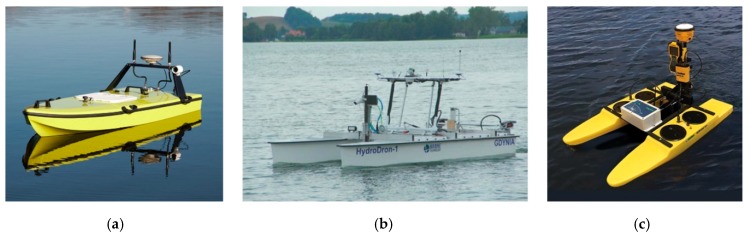
Hydrographic unmanned surface vessels. A single-hulled CEE-USV vessel (**a**), a double-hulled HydroDron (**b**), and a Seafloor Systems vessel (**c**).

The beginning of the 21st century saw the development of autonomous vessels [20,21] and the use of unmanned vessels in a variety of measurement applications [22], including hydrography. Contemporary Autonomous/Unmanned Surface Vessels (ASV/USV) are design solutions which differ from each other mainly in the construction of the hull and the type of propulsion. Single- or multi-hulled vessels with a screw or screwless propulsion are commonly used in these fields [23]. Their particular common feature is a shallow draft, which allows them to enter hard-to-reach waterbodies, including those with a shallow depth [7,24]. For this reason, the use of small unmanned vessels in both inland and marine [25] hydrography is nowadays becoming increasingly widespread (Figure 1).

**Figure 2:**
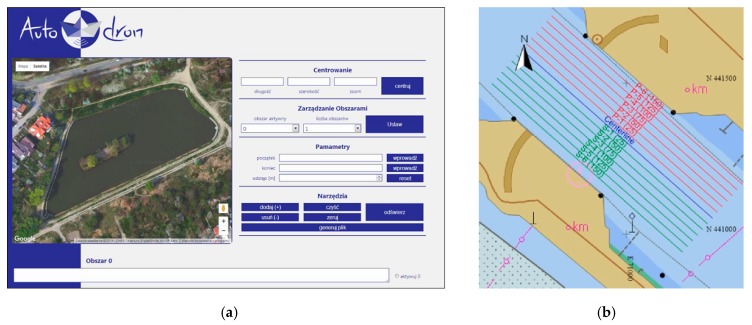
Applications used to plan sounding work, such as AutoDron (**a**) [26] and QINSy (**b**).

Small unmanned vessels, including hydrographic ones, are most often controlled either directly using a radio controller or automatically using an autopilot. For unmanned vessels, measurements are planned with software, which enables the design of sounding profiles (directions and distances between them) based on a specific measurement waterbody—usually on the basis of an orthophotomap from the Google Maps service (Figure 2a). Some of them also have a vessel position real-time monitoring capability. However, one should note that software of this type is used only by highly advanced structures of hydrographic unmanned vessels (i.e., those with an autonomous navigation mode) [26]. On the other hand, when it comes to manned vessels, there are a number of programs used to display maps and to navigate during (inland and maritime) sailing and to plan them. They include free software, such as OpenCPN and Transas iSailor, and proprietary software, such as HYPACK or QINSy (Figure 2b).

**Figure 3:**
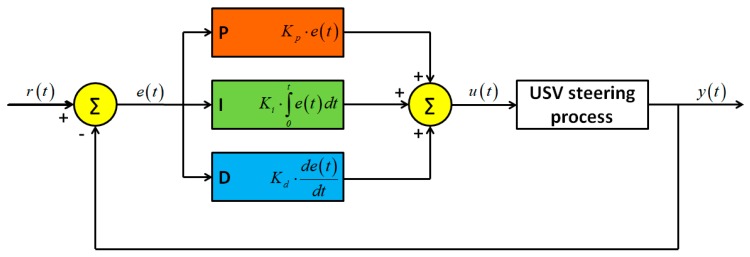
USV autopilot steering process. Own study based on [28].

Contemporary hydrographic unmanned vessels use autopilots with Proportional-Integrative-Derivative (PID) controllers, which, after the tuning process, allow the vessel to be maintained on a specified trajectory (course), referred to as a hydrographic sounding profile. A PID controller is a commonly used feedback controller, which is popular because of its simplicity in design and robustness in performance. In a PID controller, which is sometimes called a three-term controller, three separate constants work together to minimise the error between the desired commands and the actual system response [27]. The distinguishing feature of the PID controller is the ability to use the three control terms of proportional, integral, and derivative influence on the controller output to apply accurate and optimal control of the USV’s course between the points located on the sounding profile. The PID controller continuously calculates an error value, e(t), as the difference between the calculated course between the (start and end) points of the sounding profile, r(t), and the current course calculated between the current USV’s position from the Global Positioning System (GPS) and the turning point on the profile, y(t). On this basis, the autopilot calculates the control signal, u(t), as a weighted sum of the outputs of three PID control terms (Figure 3) and feeds it to the vessel steering system. The K_p_, K_i_, and K_d_ coefficients are values for determining the effect of particular terms on the automatic process control. Their values are determined in the autopilot tuning process.

**Figure 4:**
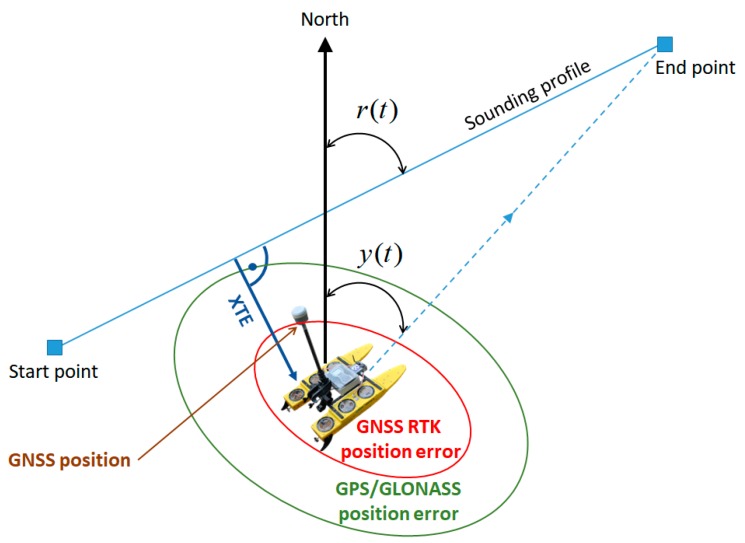
Graphic (navigation) interpretation of the automatic course adjustment system using an autopilot.

The values of variables r(t) and y(t) presented in Figure 3 are, in navigation terms, two angles. The former is the sounding profile azimuth (the angle between the line connecting the beginning and the end of the profile and the geographic north), while the latter is the current USV course, i.e., the angle between the instantaneous course line and the direction of geographic north (Figure 4).

In automatic mode, the USV autopilot should control in such a manner so that, based on the course y(t) being continuously determined, the rudder angle could be changed to obtain the end point of the profile along the shortest route. This is only possible provided that the current vessel’s position is determined using GNSS systems. Hence, the accuracy of USV positioning using a GNSS system is crucial due to the control accuracy. On the other hand, the referenced study proposed that the measure of steering precision should be the value of the transverse, lateral deviation from the course (cross track error—XTE). It should be minimised, i.e., the vessel ought to be located as close as possible to the sounding profile. For calculating XTE, the discrete location samples representing the vehicle’s path must be represented in a local Cartesian (x, y) coordinate system. While some location recording equipment may directly provide this information, others (such as that used in this work) provide output in the form of latitude and longitude coordinates. These were simply transformed using formulas and methods described in International Organization for Standardization (ISO) 12188-2:2012 standard [29]. This provided input to the XTE calculation procedure that consisted of a list of points in Cartesian coordinates for both the outbound and return paths [30]. In Figure 4, the XTE parameter, i.e., the lateral distance (transverse to the course) between the USV’s position and the sounding profile, is added.

In view of the fact that contemporary USVs use various GNSS system solutions (with various positioning accuracies) for the positioning, it is reasonable to ask: what GNSS solutions should be applied to ensure a positioning accuracy that is acceptable from the steering accuracy perspective? This study attempted to assess the accuracy of controlling a small automatic hydrographic vessel using an autopilot with a low-cost multi-GNSS receiver that is typical of recreational and Unmanned Aerial Vehicles (UAV). This solution is an alternative to the commonly applied positioning based on the GNSS geodetic network, which requires a very expensive receiver to ensure the positioning accuracy at a level of 2–5 cm (*p* = 0.95), a control system based on a marine gyrocompass or a satellite gyrocompass, and a professional vessel autopilot.

## 2. Materials and Methods

### 2.1. USV Modernisation

**Figure 5:**
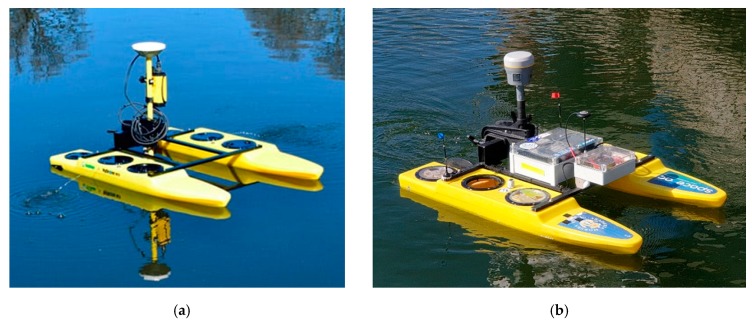
USV before (**a**) and after (**b**) the modernisation.

The first stage of research work was the technical modernisation of a hydrographic USV. The modernised USV was a small vessel controlled directly using Radio Control (RC) equipment, with no automatic mode (understood as the ability to independently accomplish a planned mission). It had a length of 1.1 m and a width of 0.7 m, with a total weight of approximately 18 kg. The USV was classified as X class, in accordance with the adopted global American nomenclature [31]. The hydrographic vessel was a commercial HyDrone vehicle manufactured by the Seafloor Systems Inc. (United States of America—USA). Its design included two hulls made from High-Density Polyethylene (HDPE) resistant to environmental conditions and mechanical factors. They were connected by an H-shaped frame made from aluminium (Figure 5a). It was normally equipped with a hydrographic system comprising a geodetic GNSS receiver (e.g., Trimble R10) and a Single Beam Echo Sounder (SBES) (e.g., SonarMite BTX). The main aims of the USV modernisation were to:

- Enable the performance of hydrographic measurement campaigns in automatic mode involving independent (with no operator’s participation) sailing along the planned sounding profiles;
- Improve the operating parameters and functionality through increasing the operating range, extending the operation time, and enhancing the reliability characteristics of both particular components and the entire system.

The USV modernisation concerned the following components: a PixHawk Cube autopilot was installed; an RC microwave transmission was modernised to increase the operation range by three times; for the autopilot control, a low-cost multi-GNSS receiver (u-blox NEO-M8N) with a built-in Fluxgate compass was used; the drive system was replaced and its power was increased (2 × 50 N); the previously used Absorbent Glass Mat (AGM) batteries (2 × 108 Wh) were replaced by LiPo batteries (2 × 326 Wh) to increase the time of operation by four times while reducing the weight by 28% (Figure 5b). Table 1 lists the basic technical characteristics and operational properties which were subject to modernisation. They were divided into two categories, related to automation (A) and operation (O).

**Figure 6:**
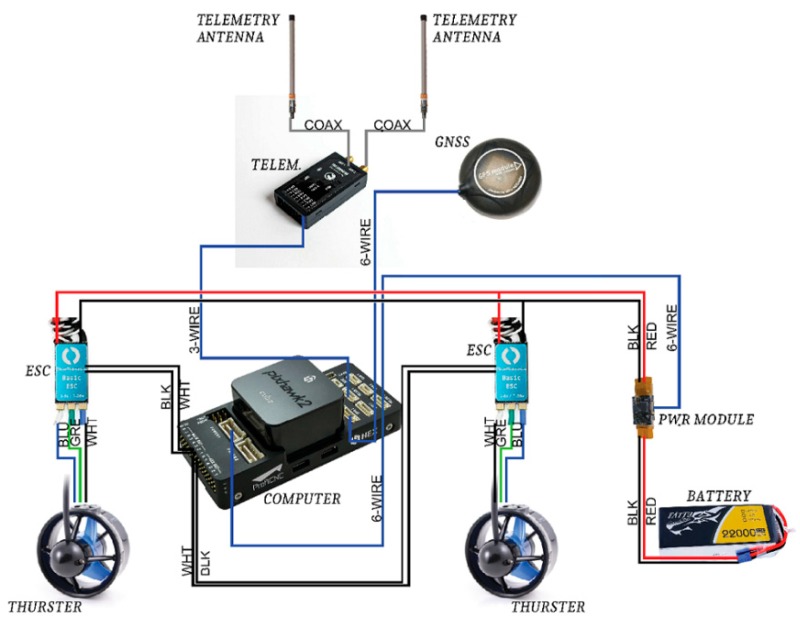
A schematic diagram of the main components subjected to the USV modernisation.

The scope of the conducted modernisation of the hydrographic USV included all its main systems: drive, control, telemetric, and positioning. The only component that was not modified was the hydrographic system, comprising a geodetic GNSS receiver and an SBES, which met the International Hydrographic Organization (IHO) requirements. Figure 6 shows the basic components subjected to modernisation and a schematic diagram of electrical connections.

### 2.2. Measurements

**Figure 7:**
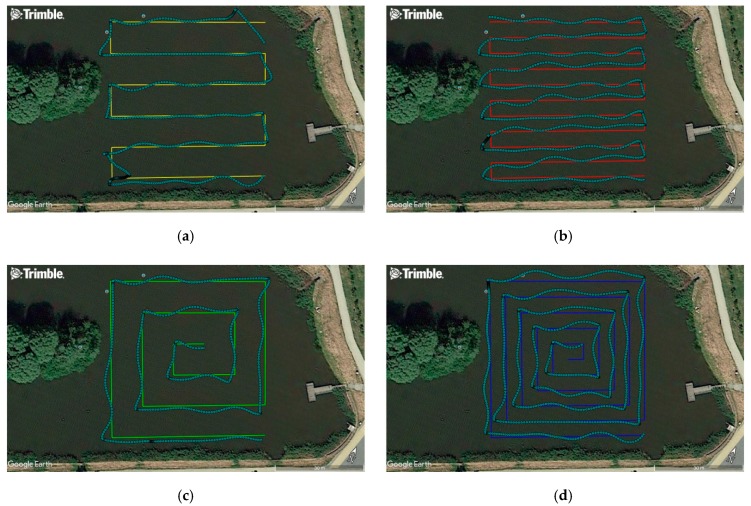
Sounding profiles designed in two variants: parallel (**a**,**b**) and spiral (**c**,**d**), along with the actual plotted USV route. Measurement points were recorded by a geodetic GNSS receiver (sky-blue colour).

The assessment of the effect of the conducted unmanned vessel modernisation on the operational properties was carried out based on experimental testing. On 7 March 2019, hydrographic measurements were performed to assess the accuracy of steering an ASV/USV in automatic mode along sounding profiles (Figure 7) designed in accordance with the principles included in the IHO S-44 standard [6]. Since the hydrometeorological conditions are an important factor affecting the obtained results, the measurements were performed in windless weather and the sea state was equal to 0 according to the Douglas scale (no wind waves or sea currents). The waterbody selected for the study was a small storage reservoir located in Gdańsk with a constant depth of 1.5 m, an orthophotomap of which was obtained from the Google Earth platform.

The study used an USV modernised to include automatic mode and equipped with a low-cost multi-GNSS receiver, whose task was to sail along the pre-set test routes. The sounding profiles were designed in four variants. For the first two routes (Figure 7a,b), they were designed in such a manner that the sounding lines were parallel to each other. The mutual distance between the profiles was assumed to be 5 and 10 m. The arrangement of the sounding profiles of two other routes (Figure 7c,d) resembled “narrowing squares” (a spiral of) towards the centre of the waterbody being sounded. The distance between the successive polygons was constant and amounted to 5 and 10 m for both variants, similar to the first two routes.

The test routes, i.e., hydrographic sounding profiles along which the USV moved, were planned using a typical geodetic software, Trimble Business Center, and satellite images obtained from the Google Earth platform. The coordinates of the route turning points were then exported to *.kml files. They were recorded as geodetic coordinates, referred to the WGS84 ellipsoid with a precision of nine decimal places. The planned USV routes set in this manner were converted to *.shp files dedicated to the ArduPilot Mission Planner software operating the 3DR PX4 Pixhawk autopilot installed on the vessel, thanks to telemetry (TBS Crossfire TX LITE).

A navigation multi-GNSS receiver NEO-M8N (GPS, GLONASS (GLObal NAvigation Satellite System), BDS (BeiDou Navigation Satellite System), Galileo, QZSS (Quasi-Zenith Satellite System), SBAS (Satellite Based Augmentation System)), which was installed along the vessel’s axis, was connected to the autopilot. In order to assess the steering accuracy, a geodetic receiver (Trimble R10) using the GNSS geodetic network was applied. The installation of two different receivers on the USV resulted from the fact that the dedicated accuracy of determining a position’s coordinates by the navigation multi-GNSS receiver was 2.5 m (CEP (Circular Error Probable), *p* = 0.50), while the geodetic receiver using the VRSNet.pl network enabled the performance of measurements in real time with an accuracy of 2–5 cm (*p* = 0.95) in both a horizontal and vertical plane. The geodetic GNSS receiver was a reference receiver based on which the XTE value was determined.

**Figure 8:**
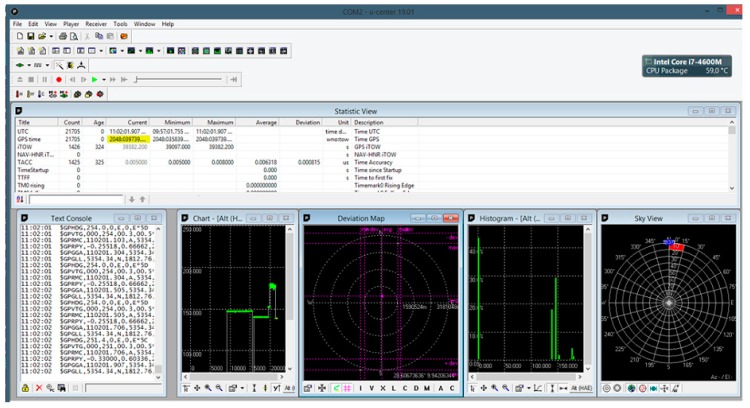
The window of the u-center software used to save messages recorded by the navigation multi-GNSS receiver in the National Marine Electronics Association (NMEA) standard.

The navigation multi-GNSS receiver was installed at the front of the mounting frame connecting both hulls of the vessel, and the geodetic receiver was placed on a 1 metre long pole attached to the rear part of the mounting frame using a special grip. The data recorded at the time of the measurements by the geodetic receiver were recorded on the controller’s internal card, while the position of the navigation receiver was sent in real time to a laptop with u-center software installed by u-blox (Figure 8).

## 3. Results

The position coordinates recorded by the geodetic GNSS receiver, i.e., the latitude and longitude, were provided in an angular (curvilinear) measurement, preventing the direct determination of the XTE value in metres, in relation to the designed sounding profiles. For this reason, the geographic (angular) coordinates were projected from the surface of the World Geodetic System 1984 (WGS84) ellipsoid (with the parameters of a = 6,378,137.00 m, b = 6,356,752.314) [32] onto a flat surface using the Gauss–Krüger transformation commonly applied in geodesy [33]. The calculations yielded two-dimensional coordinates (x, y), where the x value represents the distance (in metres) between the point and the equator, calculated along the meridian arc (on the WGS84 ellipsoid), and the variable y is the distance (in metres) to the arbitrarily set central meridian. The minus sign indicates that the point is located to the west of the meridian, while the plus sign corresponds to a location to the east of the meridian. In order to avoid negative values of the coordinates on the y axis, a constant value of, for example, 500,000 m (each zone in the PL-2000 system spanned 3° of longitude) was frequently added to the result [34,35].

The studies used the PL-2000 system, and the replacement of angular coordinates into Cartesian coordinates was conducted based on the following mathematical relationships [36]:

(1) $$\begin{array}{ll} x=m_{0}\cdot N\cdot & [\frac{S(B)}{N}+\frac{{\left(∆L\right)}^{2}}{2}\cdot \sin(B)\cdot \cos(B)+\frac{{\left(∆L\right)}^{4}}{24}\cdot \sin(B)\cdot \cos^{3}(B)\cdot (5-t^{2}+9\cdot \eta^{2}+4\cdot \eta^{4})+\\ & +\frac{{\left(∆L\right)}^{6}}{720}\cdot \sin(B)\cdot \cos^{5}(B)\cdot (61-58\cdot t^{2}+t^{4}+270\cdot \eta^{2}-330\cdot \eta^{2}\cdot t^{2}+445\cdot \eta^{4}] \end{array}$$

(2) $$\begin{array}{ll} y=m_{0}\cdot N\cdot & [∆L\cdot \cos(B)+\frac{{\left(∆L\right)}^{3}}{6}\cdot \cos^{3}(B)\cdot (1-t^{2}+\eta^{2})+\\ & +\frac{{\left(∆L\right)}^{5}}{120}\cdot \cos^{5}(B)\cdot \left(5-18\cdot t^{2}+t^{4}+14\cdot \eta^{2}-58\cdot \eta^{2}\cdot t^{2}+13\cdot \eta \right)]\\ & +500000+\frac{L_{0}}{3}\cdot 1000000 \end{array}$$

where *m*_0_ is the scale factor (-), *N* is the ellipsoid normal (radius of curvature perpendicular to the meridian) (m), *S(B)* is the meridian arc length from the equator to the arbitrary latitude (B) (m), ∆*L* is the distance between the point and the central meridian (rad), *B* and *L* are the ellipsoidal coordinates of the point (˚), and *L*_0_ is the longitude of the central meridian (˚). The other parameters of projection to the two-dimensional Cartesian coordinates in the PL-2000 system include:

(3) t=tan(B),

(4) $$\eta =\sqrt{\frac{e^{2}\cdot \cos^{2}(B)}{1-e^{2}}},$$

where *e* is the first eccentricity (-).

Then, in order to determine the XTE distances, it was necessary to describe each route profile in a mathematical manner. Since the routes were comprised of several straight sections, each profile was presented as a linear function expressed using the following formula:

(5) $$x_{i,j}=a_{i,j}\cdot y_{i,j}+b_{i,j},$$

where *i* is the route number, *j* is the section number for the *i*-th route, *x_i,j_* and *y_i,j_* are the flat rectangular coordinates PL-2000 of the point *j* recorded by a particular receiver on the *i*-th route, *a_i,j_* is a slope of the straight line *j* for the *i*-th route, defined as follows:

(6) $$a_{i,j}=\frac{x_{i,j+1}-x_{i,j}}{y_{i,j+1}-y_{i,j}},$$

*b_i,__j_* is a y-intercept of the straight line *j* for the *i*-th route, defined as follows:

(7) $$b_{i,j}=x_{i,j}-a_{i}\cdot y_{i,j}.$$

**Figure 9:**
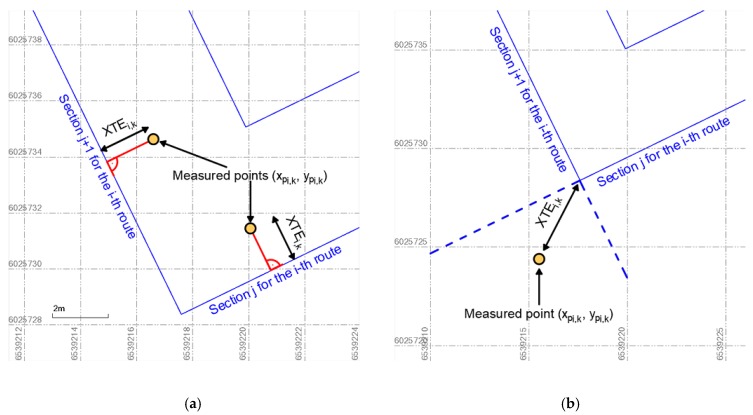
A graphical method for determining the distance of the measured point to the designed route, if the straight lines perpendicular to the hydrographic profiles could be drawn (**a**) and it is not possible to determine the perpendicular line (**b**).

The distances were then calculated between the recorded coordinates of the geodetic GNSS receiver (converted into two-dimensional x, y coordinates) and the designed route sections. During the calculations, two variants needed to be considered. The first variant assumed that for the measured points, straight lines perpendicular to the designed hydrographic profiles could be drawn (Figure 9a), while in the second variant, it was not possible to determine the perpendicular line (Figure 9b). In the first case, the XTE value was calculated using the following formula:

(8) $$XTE_{i,k}=\frac{\left|-a_{i,j}\cdot y_{p}{}_{{}_{i,k}}+x_{p}{}_{i,k}-b_{i,j}\right|}{\sqrt{{\left(-a_{i,j}\right)}^{2}+1}},$$

where *k* is the point number recorded by a particular receiver, *x_pi,k_* and *y_pi,k_* are the flat rectangular coordinates PL-2000 of the point *k* recorded by a particular receiver on the *i*-th route.

On the other hand, in the second variant the distance to the nearest route turning point was determined using the following formula:

(9) $$XTE_{i,k}=\sqrt{{\left(x_{p}{}_{i,k}-x_{i,j}\right)}^{2}+{\left(y_{p}{}_{i,k}-y_{i,j}\right)}^{2}}.$$

After calculating the XTE value, this variable was statistically analysed. Two accuracy measures were adopted as the assessment criteria: XTE (*p* = 0.68) and XTE (*p* = 0.95) which corresponded to the probabilities of 68% and 95%. Table 2 shows the values of these measures.

Based on the results presented in Table 2, it can be concluded that the statistics for the accuracy of steering an USV in the automatic mode, measured by the XTE variable for four representative routes, were very similar to each other and amounted to 0.92–1.4 m for *p* = 0.68 and 2.01–2.39 m for *p* = 0.95. The measurements demonstrated that the accuracy of maintaining the vessel along the profile was not affected by the route shape (parallel sounding lines and the “narrowing squares”) and the mutual distance between the profiles (5 and 10 m) (Figure 7).

As part of the study, an analysis of the XTE random variable distribution was conducted as well. To this end, Easy Fit software, which verifies the conformity of the measurements with typical statistical distributions using conformity tests (Kolmogorov–Smirnov, Anderson–Darling, and chi-square) was applied. The conducted analyses show that the empirical statistical distribution of the XTE values was most similar to four-parameter generalised gamma distribution with the probability density function [37]:

(10) $$f\left(XTE;\chi ,\varepsilon ,\phi \right)=\frac{\varepsilon }{\chi \cdot \Gamma \left(\phi \right)}\cdot {\left(\frac{XTE}{\chi }\right)}^{\varepsilon \cdot \phi -1}\cdot e^{-{\left(\frac{XTE}{\chi }\right)}^{\varepsilon }},$$

where *χ* is the scale parameter (*χ* > 0), *ε* is the shape parameter (*ε* > 0), and *ϕ* is the shape parameter (*ϕ* > 0), where *Γ(ϕ)* is the Euler’s Gamma function with the following form:

(11) $$\Gamma \left(\phi \right)=\int_{0}^{\infty }u^{\phi -1}\cdot e^{-u}du,$$

and the cumulative distribution function with the following form:

(12) $$F\left(XTE;\chi ,\varepsilon ,\phi \right)=\frac{\Gamma_{n}\left[\phi ,{\left(\frac{XTE}{\chi }\right)}^{\varepsilon }\right]}{\Gamma \left(\phi \right)},$$

where the introduced auxiliary variable amounts to:

(13) $$\gamma ={\left(\frac{XTE}{\chi }\right)}^{\varepsilon },$$

and the incomplete gamma function is presented by the relationship:

(14) $$\Gamma_{n}\left(\phi ,\gamma \right)=\int_{0}^{\gamma }u^{\phi -1}\cdot e^{-u}du.$$

**Figure 10:**
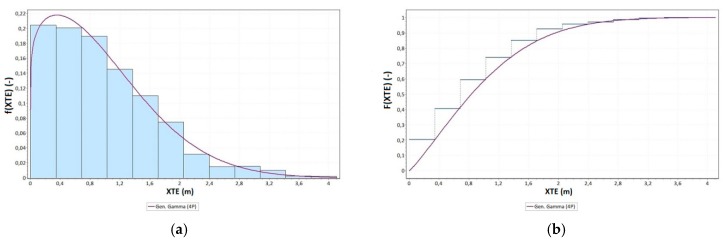
Probability density function (**a**) and cumulative distribution function (**b**) of the generalised gamma distribution of the XTE variable.

Figure 10 shows empirical and theoretical functions of the XTE variables: probability density function and cumulative distribution function.

Based on Figure 10, it can be concluded that the quantile of an order of 0.68 (percentile) in the analysed population amounted to 1.21 m, while the quantile of an order of 0.95 amounted to 2.34 m. This should be interpreted as meaning that 68% of the distance between the route recorded by geodetic GNSS receiver and the designed route was no greater than 1.21 m and, analogously, 95% of elements of this population did not exceed the value of 2.34 m. Next, the values of empirical and theoretical cumulative distribution function of the generalised gamma distribution of the XTE variable were compared using the following formula:

(15) $$∆p\left(XTE\right)=F_{n}\left(XTE\right)-F\left(XTE\right),$$

where *F_n_(XTE)* is the empirical cumulative distribution function of the XTE variable, and *F(XTE)* is the theoretical cumulative distribution function of the XTE variable.

**Figure 11:**
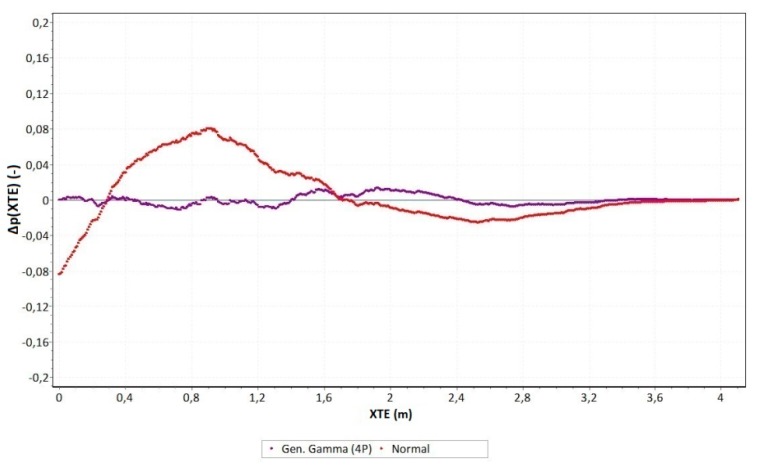
The difference between the probabilities of the occurrence of variable XTE for the empirical and theoretical generalised gamma distribution.

A graph of the Δ*p(XTE)* values is shown in Figure 11. At the same time, it was compared with the theoretical normal distribution.

Based on Figure 11, it can be observed that the empirical distribution was almost identically fitted to the theoretical generalised gamma distribution. The maximum difference between the probabilities of the occurrence of variable XTE for the empirical and theoretical generalised gamma distribution was less than ±0.01–0.015. On the other hand, for the normal (Gaussian) distribution, which was the most important probability distribution applied in practice, the analysed measure was several times greater and amounted to approximately ±0.08.

## 4. Discussion

The conducted study proved that the use of a low-cost multi-GNSS receiver as a position source for the autopilot popular in unmanned systems makes it possible to obtain a very high precision of steering for a hydrographic USV. Such a solution allows a hydrographic vessel to be maintained along the sounding profile with a transverse error (XTE) not exceeding 2 m. In traditional (manned) hydrography, sailing along sounding profiles is typically performed using systems based on a professional autopilot cooperating with a gyrocompass, satellite compass, or, less frequently, a magnetic compass. The typical accuracy of the course maintenance by gyrocompass devices is 1–1.5° [38], with that of satellite compasses being similar. However, under unfavourable conditions, it can even temporarily reach a value of 10° [39]. Moreover, expensive geodetic GNSS receivers are most frequently used in professional hydrography as a positioning system [9].

The proposed solution in this paper is an alternative to the professional measuring systems used in hydrography. The main factor affecting the accuracy of steering a hydrographic USV along a sounding profile using an autopilot is the operational characteristics of the multi-GNSS receiver. Thanks to the continuous development and extension of the GNSS satellite system constellation (GPS, GLONASS, BDS, Galileo), multi-GNSS receivers continue to improve their operational characteristics, including positioning accuracy. Many studies have attempted to verify the positioning accuracy, availability, continuity, and reliability with multi-GNSS receivers from urban [40,41,42] and maritime [43] to pedestrian positioning applications [44] using different methods [45].

It should be assumed that multi-GNSS receivers used in mobile devices will soon significantly increase their positioning accuracy thanks to the use of dual-frequency receivers [46] and other measurement techniques [40]. On 21 September 2017, Broadcom announced the world’s first mass-market, dual-frequency GNSS receiver device, the BCM47755. It is a very strong innovation destined to bring a revolution in the field of survey and geolocalisation. With these kinds of sensors, an accuracy of a few centimetres could be obtainable even with mobile devices [45]. The widespread introduction of multi-frequency GNSS receivers with an accuracy of a few centimetres is undoubtedly a major factor contributing to the improvement of unmanned vessel automatic control precision.

## 5. Conclusions

The preliminary study demonstrated that the accuracy of maintaining an USV carrying out hydrographic surveys in automatic mode using a popular Pixhawk autopilot and a multi-GNSS receiver supported by a Fluxgate magnetic compass enables the efficient performance of bathymetric measurements. The analysed solution allows a surface vessel to be maintained on sounding profiles with an accuracy of the transverse deviation from a course of 0.92–1.4 m (*p* = 0.68) and 2.01–2.39 m (*p* = 0.95). This means that it meets the IHO category 2 requirement [6] in terms of hydrographic system positioning accuracy. On this basis, it can be concluded that for the performance of selected hydrographic surveys, it is possible to use low-cost and widely available measuring equipment to successfully replace traditional solutions based on a professional autopilot. Moreover, the shallow draft of the vessel (20 cm) enables it to take bathymetric measurements of ultra-shallow waters in an automatic manner, which significantly increases their efficiency.

This publication proposed a number of modernisation solutions for a typical USV, previously controlled directly in a manual manner. It included all its basic components, such as the drive system, GNSS positioning, course control automatics, and the data transmission system. As a result, the vessel became able to take measurements in automatic mode and significantly increased its operational properties.

Research results have shown that low-cost multi-GNSS receivers can be successfully used in USVs for applications related to, among others, hydrographic surveys [47], in supporting the navigation process [21], in underwater photogrammetry [48], or in geological works [49].

## Figures and Tables

**Table 1:** A summary comparison of the USV functionality parameters before and after the modernisation.

A/O	Functionality	Before Modernisation	After Modernisation
A	Control	Direct RC	Direct RC, semi-automatic, automatic
A	RC operation range	300 m (2400 MHz)	1 km (868 MHz)
A	Telemetry monitoring	Additional PC	Integrated with the RC equipment
A	Positioning system	u-blox NEO-7: 56 channels; GPS, GLONASS, Galileo, QZSS, SBAS	u-blox NEO-M8N: 72 channels, GPS, GLONASS, BDS, Galileo, QZSS, SBAS
O	Possibility for hull replacement	No	Yes
O	Type of drive	Engines, main engine shafts, screws	Integrated pushing propellers 2 × 50 N
O	Engine cooling and ESC	Yes - forced, water	Not required
O	Battery bank	2 × 9Ah AGM (2 × 108 Wh)	2 × 22 Ah LiPo (2 × 326 Wh)
O	Time of operation until battery replacement time	1.5 h	6 h
O	Vehicle weight	25 kg	18 kg
O	Ingress protection class	IP44	IP56

**Table 2:** Statistical accuracy measures (XTE) of steering an USV for particular routes.

Accuracy Measure	Route a (10 m)	Route b (5 m)	Route c (10 m)	Route d (5 m)
Route type	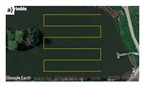	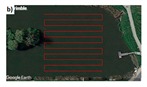	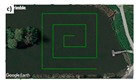	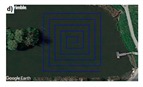
Number of measurements	458	848	506	869
XTE (*p* = 0.68)	0.92 m	1.15 m	1.40 m	1.27 m
XTE (*p* = 0.95)	2.01 m	2.38 m	2.20 m	2.39 m
